# All-cause mortality and the risk of stroke with selective aspiration thrombectomy in patients with ST-elevation myocardial infarction undergoing primary percutaneous coronary intervention: A nationwide retrospective cohort study

**DOI:** 10.1097/MD.0000000000019590

**Published:** 2020-05-29

**Authors:** Ying-Chang Tung, Lai-Chu See, Shu-Hao Chang, Hui-Tzu Tu, Yi-Hsin Chan, Chi-Jen Chang

**Affiliations:** a Cardiovascular Department, Linkou Chang Gung Memorial Hospital; b College of Medicine; c Department of Public Health, College of Medicine; d Biostatistics Core Laboratory, Molecular Medicine Research Center, Chang Gung University; e Division of Rheumatology, Allergy and Immunology, Department of Internal Medicine, Linkou Chang Gung Memorial Hospital, Taoyuan, Taiwan.

**Keywords:** mortality, physician volume, ST-elevation myocardial infarction, stroke, thrombectomy

## Abstract

Few data exist on the clinical outcomes of selective aspiration thrombectomy during primary percutaneous coronary intervention (PPCI).

This was a nationwide retrospective cohort study. Patients who were diagnosed with ST-elevation myocardial infarction (STEMI) and received primary percutaneous coronary intervention (PCI) from July 2009 to December 2011 were identified from the National Health Insurance Research Database of Taiwan. Propensity score weighting was used to balance the covariates between the 2 study groups. The primary endpoints were all-cause mortality and stroke during hospitalization and at 30 days and 1 year of follow-up. Subgroup analyses were performed based on the hospital and physician volume of primary PCI.

A total of 9100 ST-elevation myocardial infarction patients (29.4% of patients receiving aspiration thrombectomy and conventional PPCI vs 70.6% receiving PPCI alone) were identified. The incidence rates of all-cause mortality were comparable between the 2 groups during hospitalization (21.0 vs 27.37/100 person-months; *P* = .29) and 1-year follow-up (0.81 vs 1.26/100 person-months; *P* = .85). There were no significant differences in the stroke rates between the 2 groups during hospitalization (1.1 vs 2.34/100 person-months; *P* = .3) and 1-year follow-up (0.09 vs 0.15/100 person-months; *P* = .85). For the patients who survived to discharge, the post-discharge 1-year mortality was lower in the aspiration thrombectomy group of patients in whom the procedures were performed by physicians with a high volume of PPCI (hazard ratio: 0.47; 95% confidence interval: 0.24–0.94; *P* = .03).

In this nationwide cohort study, selective aspiration thrombectomy at the operation's discretion had a comparable mortality rate compared with PCI alone and did not increase the risk of stroke. In the patients treated by physicians with a high volume of PPCI, aspiration thrombectomy appeared to have a beneficial effect on post-discharge survival at 1 year.

## Abstract 2

Supplemental Digital Content is available in the text

## 1 Introduction

Primary percutaneous coronary intervention (PPCI) combined with pharmacological treatment is the cornerstone of management for patients with ST-elevation myocardial infarction (STEMI) to restore normal coronary flow and salvage jeopardized myocardium.^[1,2]^ Despite the ability of percutaneous coronary intervention (PCI) to restore patency of infarct-related arteries, adequate myocardial reperfusion may not always be achieved in patients with a high thrombus burden.^[3]^ Distal embolization during PCI may result in microvascular obstruction and impaired myocardial blush, leading to a large infarct size and consequently an unfavorable prognosis.^[4–7]^ Therefore, the use of thrombus aspiration during PPCI would seem to be a reasonable approach. The TAPAS trial (Thrombus Aspiration during PCI in Acute Myocardial Infarction Study) reported that adjunctive thrombus aspiration decreased all-cause and cardiovascular mortality rates at 1 year, and tended to reduce the risk of stent thrombosis compared with conventional PCI.^[8]^ However, 2 larger randomized control trials, the TASTE (Thrombus Aspiration in STEMI in Scandinavia) and TOTAL (Trial of Routine Aspiration Thrombectomy with PCI versus PCI Alone in Patients with STEMI) studies, found no benefits of aspiration thrombectomy in reducing the risk of all-cause or cardiovascular mortality, recurrent myocardial infarction, or stent thrombosis.^[9,10]^ In the TOTAL study, aspiration thrombectomy was even found to be associated with increased stroke rates at 30 days and 1 year.^[10,11]^

In contrast to the required assignment of treatment options in a randomized control trial, the real-world application of aspiration thrombectomy is more selective, and is based on the physician's judgment of thrombus burden, coronary flow, and specific anatomy of the target lesions. Therefore, the clinical outcomes may be different between the selective use of thrombectomy and its use in randomized control trials. In addition, the prognosis of PPCI has been demonstrated to be associated with hospital and physician volumes.^[12–15]^ In the present study, we aimed to investigate the effect of the selective use of aspiration thrombectomy on the risks of all-cause mortality and stroke in patients with STEMI treated with PPCI, as well as the impact of hospital and physician volume of PPCI on the clinical outcomes of aspiration thrombectomy.

## 2 Methods

### 2.1 Data source

The National Health Insurance Program is a government-run, mandatory health insurance program that currently covers approximately 99% of the population in Taiwan. The National Health Insurance Research Database (NHIRD) contains claims data for reimbursement, including outpatient care and inpatient registries from all medical facilities. This database provides patient-level data on sociodemographic information, dates of admission and discharge, diagnoses, prescription drugs, and the use of medical equipment. The accuracy of the NHIRD with regards to the diagnoses of myocardial infarction and stroke as well as mortality associated with these events has been validated in several studies.^[16–18]^ The Institutional Review Board of Linkou Chang Gung Memorial Hospital approved this study (No. 104-2932B).

### 2.2 Study population

**Figure 1:**
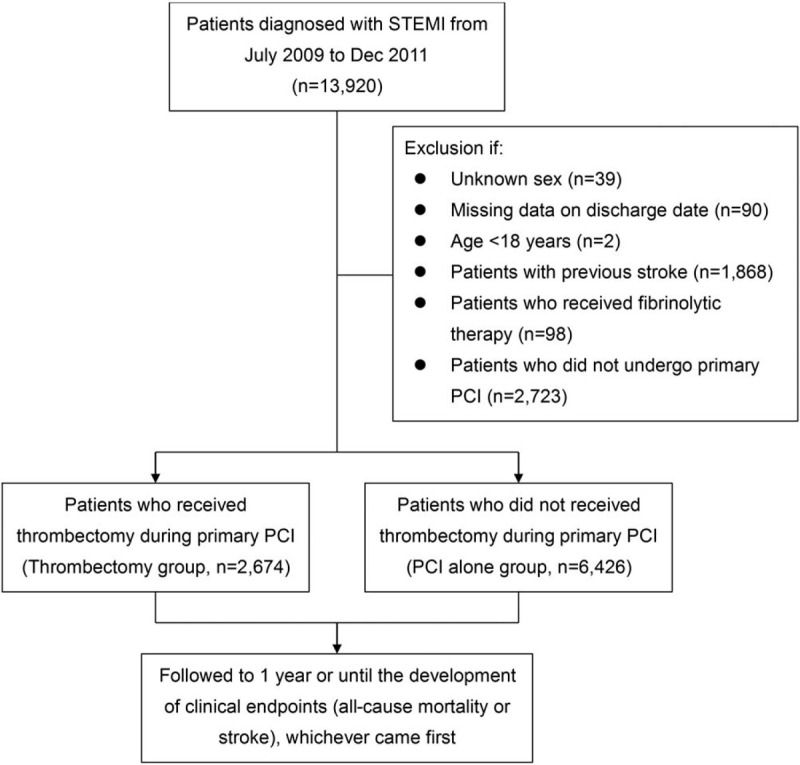
Patient enrollment and study design. PCI = percutaneous coronary intervention, STEMI = ST-elevation myocardial infarction.

Patients who were admitted due to a new diagnosis of STEMI and who were treated with PPCI from July 2009 to December 2011 were enrolled in this study. This period was chosen because the NHI program started to reimburse costs for thrombectomy devices in July 2009. STEMI was identified according to International Classification of Diseases, 9th Revision, Clinical Modification (ICD-9-CM) diagnosis codes 410.0x-410.6x or 410.8x.^[19]^Figure 1 illustrates the flowchart of patient enrollment. We excluded patients with missing data on sex (n = 39) or discharge date (n = 90), and those aged less than 18 years (n = 2), with previous stroke (n = 1,868), who received thrombolytic therapy (n = 98), and did not undergo PPCI (n = 2,723). The reason for excluding patients with a history of stroke before the diagnosis of STEMI is to avoid misclassification, because some clinicians may just copy the previous diagnosis for the purpose of reimbursement even if no new stroke event occurs during follow-up. Therefore, the stroke events were all new events in this study and should not confound by previous strokes or misclassification bias.

### 2.3 Study design

We conducted a nationwide retrospective cohort study using the NHIRD of Taiwan. The STEMI patients who were selected for aspiration thrombectomy at the operator's discretion during PPCI were defined as the thrombectomy group, and the remaining patients were defined as the PCI alone group. We defined the date of STEMI onset as the index date. ICD-9-CM codes were used to identify underlying comorbidities. Data regarding the prescription of in-hospital and outpatient medications and the utilization of medical devices were also extracted from the NHIRD using ICD-9-CM procedure codes and pharmacology and device codes. All patients were followed for 1 year or until the outcomes were achieved, whichever came first.

### 2.4 Study outcomes

The primary endpoints of this study were all-cause mortality and stroke. We analyzed the endpoints in 3 time intervals:

(1) during the index hospitalization;
(2) 30 days and 1year post-discharge; and
(3) overall at 30 days and 1 year from the index date.

Ischemic and hemorrhagic subtypes of stroke were further identified according to ICD-9-CM codes. To avoid misclassification, the diagnosis of an ischemic or hemorrhagic stroke was confirmed according to:

(1) a diagnosis at the index or subsequent hospitalizations, or
(2) a diagnosis in outpatient records after discharge.

### 2.5 Statistical analysis

Continuous variables were expressed as mean ± standard deviation, and categorical variables were expressed as frequency and percentage. Propensity score weighting was used to reduce potential differences between the 2 study groups.^[21,22]^ In this study, the propensity score was defined as the probability of a patient receiving aspiration thrombectomy during PPCI. All the baseline characteristics in Table 1, including the patient's age, sex, hospital PPCI volume, physician PPCI volume, comorbidities, management, and medications, were included in the logistic regression to obtain the propensity score. The inverse probability of treatment weighting of propensity scores was used to estimate the average treatment effect for the treated (ATT) by balancing covariates between the aspiration thrombectomy and the PCI alone groups. In the inverse probability of treatment weighting for ATT, the treated group (thrombectomy) was given a weight of 1 and the control group (PCI alone) was given weight of p/(1–p), where p is the probability of receiving thrombectomy. Hence, the thrombectomy group maintained the original sample size and the distribution of covariates in Table 1. The PCI alone group was resembled as the thrombectomy group regarding to the sample size and the distribution of covariates. An absolute standardized mean difference of less than 0.1 was taken to indicate a negligible difference between the 2 groups.^[23]^ The incidence rates of all-cause mortality and stroke were estimated as the total number of events during the follow-up period divided by the person-months at risk. For all-cause mortality, a Cox proportional hazard model was used to obtain hazard ratios (HRs). For stroke, we used Fine and Gray's competing-risk regression, which accounts for the effect of death, to obtain sub-hazard ratios (SHRs).^[24]^ The cumulative incidence of stroke versus follow-up time was plotted rather than the event-free rate, because cumulative incidence function can take the competing risk of death into account. For the thrombectomy group, 95% confidence intervals (CIs) of the HRs and SHRs were calculated using the PCI alone group as the referent group.

**Table 1:**
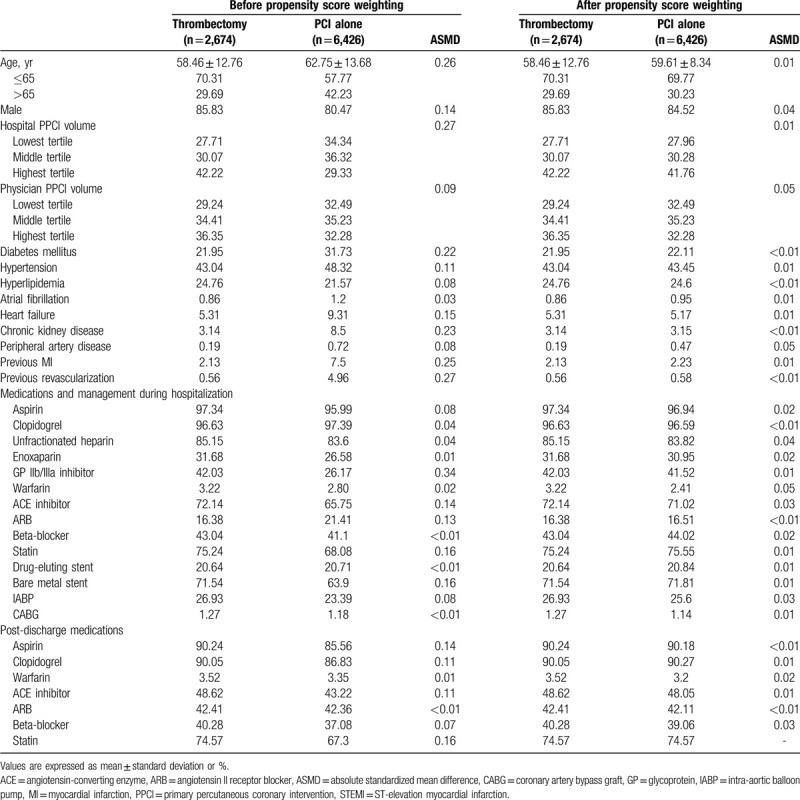
Demographic characteristics, comorbidities, and medication use of the STEMI patients treated with thrombectomy or PCI alone.

We analyzed the data in 3 parts:

(1) during the index hospitalization;
(2) 30 days and 1year post-discharge; and
(3) overall at 30 days and 1 year from the index date.

For the analysis of the outcomes of the index hospitalization, the medications used after discharge were not included when calculating the propensity score. Furthermore, we only counted the study outcomes up to 30 days during the index hospitalization, as only a very small number of patients stayed in the hospital for more than 30 days (Supplemental Digital Content [Table S1, http://links.lww.com/MD/E254]). To analyze post-discharge outcomes, we excluded the patients who died or had a stroke during the index hospitalization, and recalculated the propensity score (Supplemental Digital Content [Table S2, http://links.lww.com/MD/E255]). We hypothesized that hospital volume and physicians’ experience in PPCI may also affect the outcomes of aspiration thrombectomy. Therefore, we performed subgroup analysis based on tertiles of hospital and physician volume of PPCI. We recalculated the propensity score weighting to ensure balance between the 2 study groups in the subgroup analysis. A *P* value of less than .05 was considered to indicate statistical significance. All statistical analyses were performed using SAS version 9.4 (SAS Institute Inc., Cary, NC).

## 3 Results

From July 2009 to December 2011, 9,100 STEMI patients underwent PPCI and were eligible for inclusion into this study. Among them, 2,674 (29.4%) received aspiration thrombectomy during PPCI, and 6,426 (70.6%) received PCI alone, suggesting that thrombectomy was performed selectively. After propensity score weighting, all of the values of absolute standardized mean differences were <0.1, indicating that the 2 study groups were well balanced regarding all variables (Table 1).

**Figure 2:**
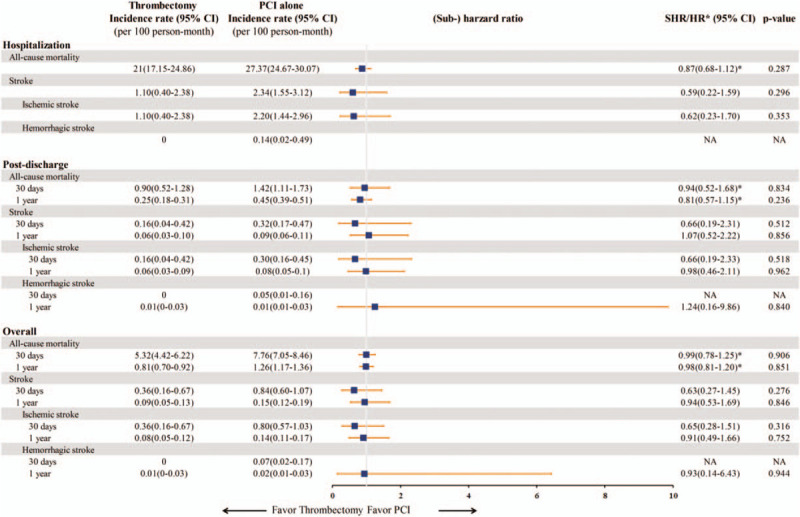
The incidence rates and (sub-)hazard ratios of all-cause mortality and stroke in the STEMI patients treated with thrombectomy or PCI alone during the index hospitalization, post-discharge and overall study period. CI = confidence interval; HR = hazard ratio, NA = not applicable because of zero events, PCI = percutaneous coronary intervention, SHR = sub-hazard ratio, STEMI = ST-elevation myocardial infarction.

**Figure 3:**
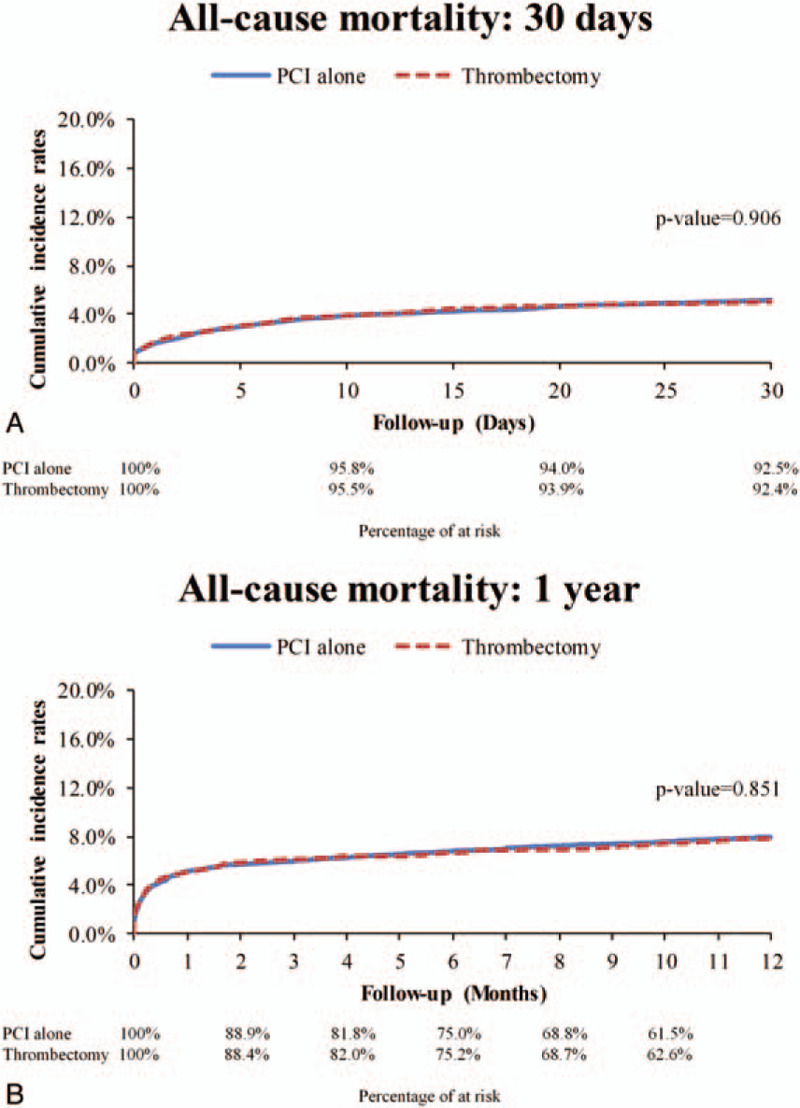
Cumulative incidence rates of all-cause mortality in the STEMI patients treated with thrombectomy or PCI alone. PCI = percutaneous coronary intervention; STEMI = ST-elevation myocardial infarction.

The median duration of hospitalization was 5 days, and the mean duration of hospitalization was 7.65 ± 4.86 days. During the index hospitalization, there was no significant difference in all-cause mortality between the 2 study groups, with incidence rates of 21.0 per 100 person-months for the thrombectomy group and 27.37 per 100 person-months for the PCI alone group (HR: 0.87; 95% CI: 0.68 to 1.12; *P* = .29) (Fig. 2). The incidence rates of all-cause mortality at 30 days were 5.32 and 7.76 per 100 person-months, respectively (HR: 0.99; 95%: CI 0.78 to 1.25, *P* = .91). At 1 year of follow-up, there was still no significant difference in all-cause mortality between the 2 groups (0.81 vs 1.26 per 100 person-months; HR: 0.98; 95% CI: 0.81 to 1.2, *P* = .85) (Figs. 2 and 3).

**Figure 4:**
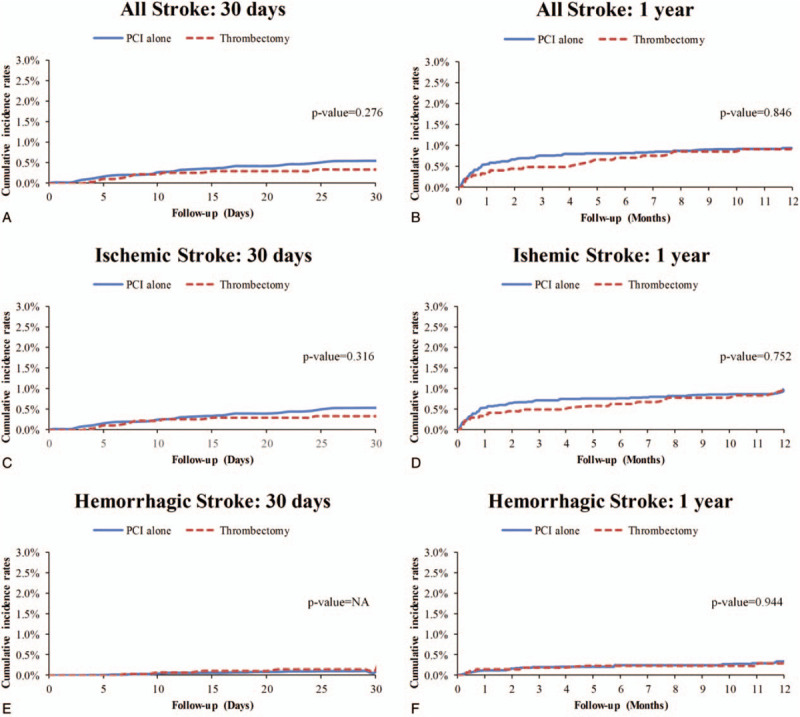
Cumulative incidence rates of stroke in the STEMI patients treated with thrombectomy or PCI alone.NA = not applicable because of zero events, PCI = percutaneous coronary intervention, STEMI = ST-elevation myocardial infarction.

The incidence rates of stroke during the index hospitalization were 1.1 and 2.34 per 100 person-months in the thrombectomy and PCI alone groups, respectively (SHR: 0.59; 95% CI: 0.22 to 1.59; *P* = .3) (Fig. 2). The incidence of stroke did not differ significantly between the 2 groups at 30 days (0.36 vs 0.84 per 100 person-months; SHR: 0.63; 95% CI: 0.27 to 1.45; *P* = .28) or 1 year of follow-up (0.09 vs 0.15 per 100 person-months; SHR: 0.94, 95% CI: 0.53 to 1.69; *P* = .85). In addition, the incidence rates of ischemic and hemorrhagic stroke were comparable between the 2 groups (Figs. 2 and 4). After excluding the patients who died or had a stroke during the index hospitalization, post-discharge outcomes were comparable with the results of the main analysis, and no significant differences were detected in all-cause mortality or stroke between the 2 groups (Fig. 2).

**Figure 5:**
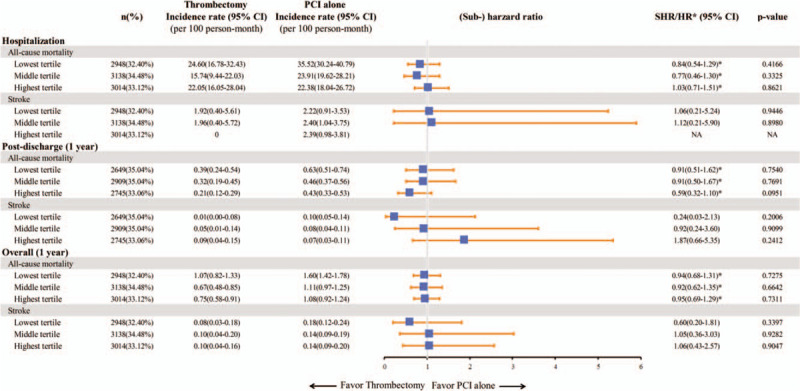
Impact of hospital volume of primary PCI on the outcomes of the patients with STEMI treated with aspiration thrombectomy vs. PCI alone. HR = hazard ratio, NA = not applicable because of zero events, PCI = percutaneous coronary intervention, SHR = sub-hazard ratio, STEMI = ST-elevation myocardial infarction.

**Figure 6:**
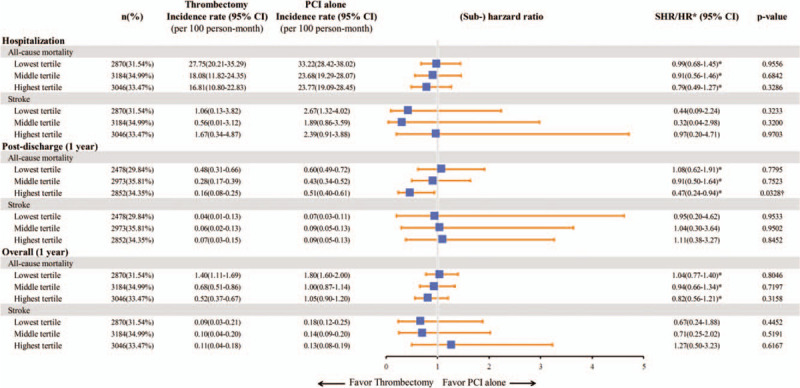
Impact of physician volume of primary PCI on the outcomes of the patients with STEMI treated with aspiration thrombectomy vs. PCI alone. HR = hazard ratio, PCI = percutaneous coronary intervention, SHR = sub-hazard ratio, STEMI = ST-elevation myocardial infarction. † Denotes *P* < .05.

In the subgroup analysis based on the hospital and physician volume of PPCI, there were no significant differences in the outcomes of aspiration thrombectomy versus PCI alone during hospitalization or at 1 year of follow-up. However, when the patients who died or had a stroke during hospitalization were excluded, the patients treated with aspiration thrombectomy at hospitals with a high PPCI volume tended to have a lower post-discharge mortality rate at 1 year compared with those receiving PCI alone (HR: 0.59; 95% CI: 0.32 to 1.1; *P* = .1; Fig. 5 and Supplemental Digital Content [Table S3, http://links.lww.com/MD/E256]). Furthermore, the post-discharge mortality rate at 1 year was found to be lower in the subgroup of patients treated with aspiration thrombectomy by physicians with a high PPCI volume (HR: 0.47; 95% CI: 0.24 to 0.94; *P* = .03; Fig. 6 and Supplemental Digital Content [Table S4, http://links.lww.com/MD/E257]).

## 4 Discussion

This retrospective nationwide population-based study investigated the real-world outcomes of the selective use of aspiration thrombectomy during PPCI. With this selective strategy based on the physician's judgment, no significant differences were detected between the thrombectomy and PCI alone groups in terms of all-cause mortality and stroke during hospitalization or at 30 days and 1 year of follow-up. In the subgroups of patients treated by physicians with a high PPCI volume, aspiration thrombectomy resulted in a better post-discharge survival at 1 year compared with PCI alone.

Previous large randomized trials have failed to demonstrate clinical benefits from unselective aspiration thrombectomy.^[9,10]^ Neutral results in randomized controlled trials can be sometimes explained by the “quantitative interaction,” the principle that patients who are at high risk of adverse outcomes tend to have the maximum benefit from a given treatment.^[25]^ In a selected cohort of STEMI patients who had angiographic evidence of high thrombus burden, De Rosa et al demonstrated that rheolytic thrombectomy improved angiographic results and was associated with better long-term outcomes compared with conventional PCI alone.^[26]^ In contrast to the assignment of thrombectomy required in randomized controlled trials, the strategy used in real-world practice usually selects patients with large thrombus burden. In our study, fewer than 30% of the patients were judged by the physicians to require thrombectomy during PPCI. With this highly selective strategy, there was no significant difference in the incidence rates of all-cause mortality between those judged to require thrombectomy and those judged to require PCI alone. Similar results were reported in an observational cohort study of the British Cardiovascular Intervention Society using the same selective thrombectomy strategy.^[27]^ This study showed similar mortality rates at 30 days and 1 year in patients who received selective thrombectomy vs. those undergoing PCI alone. Taken together, these real-world studies showed that aspiration thrombectomy, upfront or bailout, in patients with large thrombus burden and thus potentially unfavorable prognosis resulted in comparable outcomes compared with those who were judged to require conventional PCI alone. Therefore, aspiration thrombectomy should remain an important tool in the interventional cardiologist's armamentarium.

A potentially important finding of this study is that for those who survived to discharge, aspiration thrombectomy was associated with a survival benefit at 1 year in the subgroup of patients treated by physicians with a high PPCI volume. Similarly, a trend favoring thrombectomy was found in the patients who survived to discharge and received the procedure at high-volume hospitals. However, neither the physician nor hospital volume of PPCI affected the in-hospital survival. This suggests that for patients with the highest level of disease severity, aspiration thrombectomy may not significantly affect the in-hospital outcomes. In contrast, for the patients who survive to discharge, selective aspiration thrombectomy performed by experienced physicians may have a beneficial effect on post-discharge survival. Previous studies have demonstrated a positive impact of the hospital and physician volumes of PPCI on patient outcomes.^[12–15]^ Our results emphasize that experience and expertise may be important factors to optimize the outcomes of aspiration thrombectomy in patients with STEMI. Although manual aspiration thrombectomy is not a complex procedure, effective and safe aspiration of the thrombus may require experience and skill, and therefore standardizing the procedure step by step and clearly defining the endpoints of the procedure may be important.

With respect to safety outcomes, we found that the incidence rates of overall stroke as well as those of the ischemic and hemorrhagic subtypes were comparable between the thrombectomy and PCI alone groups during hospitalization and at 30 days and 1 year of follow-up. As mentioned above, the selective strategy in a real-world setting tends to mean that the patients who receive aspiration thrombectomy have a higher thrombus burden. Nevertheless, we did not find an increased risk of stroke in the patients who underwent aspiration thrombectomy. Similarly, the aforementioned British Cardiovascular Intervention Society study showed similar stroke rates in the patients with and without thrombectomy during hospitalization, although follow-up data on the risk of stroke were not reported.^[27]^ In the TOTAL study, aspiration thrombectomy was associated with a significant increase in the stroke rate at 30 days, and this trend persisted even at 1 year.^[10,11]^ Although stroke was a pre-specified endpoint in the TOTAL study, the results should be interpreted with caution. It has been proposed that stroke associated with thrombectomy may result from dislodged aspirated thrombus debris from the tip of the aspiration catheter during withdrawal or dislodgement of atheroma from the aorta caused by manipulation of the guiding catheter, resulting in embolization in the cerebral circulation.^[28]^ Therefore, thrombectomy-associated stroke would most likely occur during PPCI or be detected early in the post-procedural period. In a landmark analysis of the TOTAL study, a significant increase in the risk of stroke was not detected in the thrombectomy group immediately after PCI (<12 hours) but at 48 hours, and there was also a trend of a higher risk of stroke in the thrombectomy group from 90 days to 1 year.^[28]^ It is unclear whether this higher risk of stroke beyond 90 days was due to chance or whether it was caused by other unmeasured confounders. The TOTAL study did not provide detailed data on the incidence of atrial fibrillation, which could predispose patients to ischemic stroke during hospitalization and follow-up.^[29,30]^ In an updated meta-analysis, aspiration thrombectomy in PPCI was associated with a nonsignificant increase in the risk of stroke (relative risk: 1.45; 95% CI: 0.96 to 2.21; *P* = .08).^[31]^ However, this result was still largely due to the TOTAL study.

This study used data from a nationwide claims database and has the inherent limitations of a retrospective study. First, the NHIRD does not contain data on the characteristics of the culprit lesions, distal embolization, myocardial blush grade, or Thrombolysis in Myocardial Infarction flow grade before and after PCI. We were unable to analyze angiographic results of thrombectomy or investigate whether angiographic surrogates translated into clinical outcomes. A meta-regression analysis of randomized trials suggests that the benefit of aspiration thrombectomy over PCI alone is higher in patients who presented with a longer ischemic time.^[25]^ However, this database does not contain data on ischemic time. Survival analysis in this study was also limited by the unavailability of left ventricular ejection fraction in our database. Second, we could not perform detailed landmark analysis using a cut-off point shorter than 24 hours, and the temporal relationship between aspiration thrombectomy and stroke could not be further determined. However, since there were no significant differences between the 2 groups in the incidence rates of stroke during the index hospitalization or during the follow-up period, we think that the selective use of aspiration thrombectomy may not be associated with a higher risk of stroke. Third, identification of baseline demographic data, comorbidities and the use of interventional devices and medications was based on the diagnostic and procedural codes registered by the interventionists and the treating physicians, and incorrect coding or under-recognition of minor complications is possible in daily clinical practice. Although the risk factors for mortality and stroke were well balanced between the 2 groups using the propensity score method, there may have been confounders that were not identified in the database or adjusted by the statistical methods. Nevertheless, the accuracy of the diagnoses of myocardial infarction and stroke and subsequent mortality associated with these events has been well validated in several studies using data from the NHIRD.^[16–18]^ Therefore, the endpoints of mortality and stroke in this study should be reliable. Lastly, to avoid misclassification, the clinical endpoints did not include recurrent MI because some doctors may just copy the diagnosis of index MI for the purpose of reimbursement even if no new infarct occurs during follow-up.

## 5 Conclusions

In this nationwide database analysis, the STEMI patients who were selected for aspiration thrombectomy at the physician's discretion during PPCI had an all-cause mortality rate comparable to those who underwent PCI alone. In contrast to the results of recent large randomized control trials, the selective use of aspiration thrombectomy in the real-world setting of this study did not increase the risk of stroke during hospitalization or at 30 days and 1 year of follow-up. For the patients who survived to discharge, selective aspiration thrombectomy performed by experienced physicians with a high PPCI volume may have a beneficial effect on post-discharge mortality at 1 year.

## Author contributions

**Conceptualization:** Ying-Chang Tung, Chi-Jen Chang.

**Data curation:** Lai-Chu See, Shu-Hao Chang, Hui-Tzu Tu, Yi-Hsin Chan.

**Formal analysis:** Ying-Chang Tung, Lai-Chu See, Shu-Hao Chang, Hui-Tzu Tu.

**Funding acquisition:** Chi-Jen Chang.

**Investigation:** Ying-Chang Tung, Lai-Chu See, Shu-Hao Chang, Hui-Tzu Tu, Yi-Hsin Chan.

**Methodology:** Lai-Chu See, Yi-Hsin Chan, Chi-Jen Chang.

**Project administration:** Ying-Chang Tung.

**Resources:** Chi-Jen Chang.

**Supervision:** Lai-Chu See, Chi-Jen Chang.

**Writing – original draft:** Ying-Chang Tung.

**Writing – review & editing:** Ying-Chang Tung, Lai-Chu See, Chi-Jen Chang.

## Supplementary Material

SUPPLEMENTARY MATERIAL
