# Supplementary material for: All-cause mortality and the risk of stroke with selective aspiration thrombectomy in patients with ST-elevation myocardial infarction undergoing primary percutaneous coronary intervention: A nationwide retrospective cohort study
Source: Medicine (Baltimore). 2020 May 29;99(22):e19590. doi: 10.1097/MD.0000000000019590 (PMC12245257; doi:10.1097/MD.0000000000019590)
Supplement: SUPPLEMENTARY MATERIAL [file medi-99-e19590-s001.docx]

Table S1. Demographic characteristics, comorbidities, and medication use of the STEMI patients treated with thrombectomy or PCI alone (excluding the post-discharge medications from propensity score weighting)

|  | Before propensity score weighting | | |  | After propensity score weighting | | | | |
| --- | --- | --- | --- | --- | --- | --- | --- | --- | --- |
|  | Thrombectomy | PCI alone |  |  | Thrombectomy | | | PCI alone |  |
|  | (n=2,674) | (n=6,426) | ASMD |  | (n=2,674) | | | (n=6,426) | ASMD |
| Age, years | 58.46±12.76 | 62.75±13.68 | 0.264 |  | 58.46±12.76 | | | 59.64±8.38 | 0.013 |
| ≤65 | 70.31 | 57.77 |  |  | 70.31 | | | 69.73 |  |
| >65 | 29.69 | 42.23 |  |  | 29.69 | | | 30.27 |  |
| Male | 85.83 | 80.47 | 0.144 |  | 85.83 | | | 84.53 | 0.037 |
| Hospital PPCI volume |  |  | 0.272 |  |  | | |  | 0.008 |
| Lowest tertile | 27.71 | 34.34 |  |  | 27.71 | | | 27.95 |  |
| Middle tertile | 30.07 | 36.32 |  |  | 30.07 | | | 30.21 |  |
| Highest tertile | 42.22 | 29.33 |  |  | 42.22 | | | 41.84 |  |
| Physician PPCI volume |  |  | 0.092 |  |  | | |  | 0.046 |
| Lowest tertile | 29.24 | 32.49 |  |  | 29.24 | | | 29.99 |  |
| Middle tertile | 34.41 | 35.23 |  |  | 34.41 | | | 35.82 |  |
| Highest tertile | 36.35 | 32.28 |  |  | 36.35 | | | 34.19 |  |
| Diabetes mellitus | 21.95 | 31.73 | 0.222 |  | 21.95 | | | 22.13 | 0.004 |
| Hypertension | 43.04 | 48.32 | 0.106 |  | 43.04 | | | 43.45 | 0.008 |
| Hyperlipidemia | 24.76 | 21.57 | 0.076 |  | 24.76 | | | 24.48 | 0.007 |
| Atrial fibrillation | 0.86 | 1.20 | 0.034 |  | 0.86 | | | 0.97 | 0.011 |
| Heart failure | 5.31 | 9.31 | 0.154 |  | 5.31 | | | 5.17 | 0.006 |
| Chronic kidney disease | 3.14 | 8.50 | 0.23 |  | 3.14 | | | 3.15 | 0.001 |
| Peripheral artery disease | 0.19 | 0.72 | 0.079 |  | 0.19 | | | 0.46 | 0.048 |
| Previous MI | 2.13 | 7.50 | 0.253 |  | 2.13 | | | 2.22 | 0.006 |
| Previous revascularization | 0.56 | 4.96 | 0.271 |  | 0.56 | | | 0.58 | 0.003 |
| **Medications and management during hospitalization** | | | | | | | | | |
| Aspirin | 97.34 | 95.99 | 0.076 | | |  | 97.34 | 96.65 | 0.041 |
| Clopidogrel | 96.63 | 97.39 | 0.044 | | |  | 96.63 | 96.56 | 0.004 |
| Unfractionated heparin | 85.15 | 83.60 | 0.043 | | |  | 85.15 | 83.89 | 0.035 |
| Enoxaparin | 31.68 | 26.58 | 0.112 | | |  | 31.68 | 31.07 | 0.013 |
| GP IIb/IIIa inhibitor | 42.03 | 26.17 | 0.339 | | |  | 42.03 | 41.54 | 0.010 |
| Warfarin | 3.22 | 2.80 | 0.024 | | |  | 3.22 | 2.45 | 0.046 |
| ACE inhibitor | 72.14 | 65.75 | 0.138 | | |  | 72.14 | 70.47 | 0.037 |
| ARB | 16.38 | 21.41 | 0.129 | | |  | 16.38 | 16.49 | 0.003 |
| Beta blocker | 43.04 | 41.10 | 0.039 | | |  | 43.04 | 43.81 | 0.015 |
| Statin | 75.24 | 68.08 | 0.159 | | |  | 75.24 | 75.54 | 0.007 |
| Drug-eluting stent | 20.64 | 20.71 | 0.002 | | |  | 20.64 | 20.85 | 0.005 |
| Bare metal stent | 71.54 | 63.90 | 0.164 | | |  | 71.54 | 71.81 | 0.006 |
| IABP | 26.93 | 23.39 | 0.082 | | |  | 26.93 | 26.11 | 0.019 |
| CABG | 1.27 | 1.18 | 0.008 | | |  | 1.27 | 1.22 | 0.005 |
| Values are expressed as mean ± standard deviation or %.  ACE = angiotensin-converting enzyme; ARB = angiotensin II receptor blocker; ASMD = absolute standardized mean difference; CABG = coronary artery bypass graft; GP = glycoprotein; IABP = intra-aortic balloon pump; MI = myocardial infarction; PPCI = primary percutaneous coronary intervention; STEMI = ST-elevation myocardial infarction. | | | | | | | | | |
