# Supplementary material for: All-cause mortality and the risk of stroke with selective aspiration thrombectomy in patients with ST-elevation myocardial infarction undergoing primary percutaneous coronary intervention: A nationwide retrospective cohort study
Source: Medicine (Baltimore). 2020 May 29;99(22):e19590. doi: 10.1097/MD.0000000000019590 (PMC12245257; doi:10.1097/MD.0000000000019590)
Supplement: SUPPLEMENTARY MATERIAL [file medi-99-e19590-s002.docx]

Table S2. Demographic characteristics, comorbidities, and medication use of the STEMI patients who did not die or have a stroke during hospitalization (excluding the medications and management during hospitalization from propensity score weighting)

|  | Before propensity score weighting | | |  | After propensity score weighting | | |
| --- | --- | --- | --- | --- | --- | --- | --- |
|  | Thrombectomy | PCI alone |  |  | Thrombectomy | PCI alone |  |
|  | (n=2,500) | (n=5,803) | ASMD |  | (n=2,500) | (n=5,803) | ASMD |
| Age | 57.89±12.47 | 61.82±13.39 | 0.235 |  | 57.89±12.47 | 59.13±8.4 | 0.006 |
| ≤65 | 71.88 | 60.87 |  |  | 71.88 | 71.6 |  |
| >65 | 28.12 | 39.13 |  |  | 28.12 | 28.4 |  |
| Male | 86.36 | 81.97 | 0.12 |  | 86.36 | 85.09 | 0.036 |
| Hospital PPCI volume |  |  | 0.272 |  |  |  | 0.003 |
| Lowest tertile | 27.44 | 33.83 |  |  | 27.44 | 27.41 |  |
| Middle tertile | 30.76 | 36.88 |  |  | 30.76 | 30.88 |  |
| Highest tertile | 41.8 | 29.3 |  |  | 41.8 | 41.71 |  |
| Physician PPCI volume |  |  | 0.092 |  |  |  | 0.026 |
| Lowest tertile | 27.92 | 30.67 |  |  | 27.92 | 29.03 |  |
| Middle tertile | 35.12 | 36.1 |  |  | 35.12 | 34.96 |  |
| Highest tertile | 36.96 | 33.22 |  |  | 36.96 | 36.01 |  |
| Diabetes mellitus | 21.64 | 30.88 | 0.211 |  | 21.64 | 21.76 | 0.003 |
| Hypertension | 43.04 | 48.27 | 0.105 |  | 43.04 | 43.44 | 0.008 |
| Hyperlipidemia | 25.84 | 23.01 | 0.066 |  | 25.84 | 25.27 | 0.013 |
| Atrial fibrillation | 0.8 | 1.10 | 0.031 |  | 0.8 | 0.95 | 0.016 |
| Heart failure | 5.24 | 8.74 | 0.138 |  | 5.24 | 5.09 | 0.007 |
| Chronic kidney disease | 2.96 | 7.53 | 0.206 |  | 2.96 | 2.96 | <0.001 |
| Peripheral artery disease | 0.16 | 0.67 | 0.08 |  | 0.16 | 0.43 | 0.05 |
| Previous MI | 2.0 | 7.58 | 0.264 |  | 2.0 | 2.08 | 0.006 |
| Previous revascularization | 0.48 | 5.14 | 0.285 |  | 0.48 | 0.49 | 0.002 |
| **Post-discharge medications** | | | | | | | |
| Aspirin | 95.04 | 92.83 | 0.093 |  | 95.04 | 94.24 | 0.036 |
| Clopidogrel | 94.64 | 94.00 | 0.029 |  | 94.68 | 95.05 | 0.017 |
| Warfarin | 3.68 | 3.41 | 0.015 |  | 3.68 | 3.1 | 0.032 |
| ACE inhibitor | 51.52 | 47.17 | 0.087 |  | 51.52 | 49.53 | 0.04 |
| ARB | 44.88 | 46.11 | 0.025 |  | 44.88 | 43.93 | 0.019 |
| Beta-blocker | 42.44 | 40.12 | 0.047 |  | 42.44 | 40.98 | 0.03 |
| Statin | 79.0 | 73.29 | 0.134 |  | 79.0 | 79.23 | 0.006 |
| Values are expressed as mean ± standard deviation or %.  ACE = angiotensin-converting enzyme; ARB = angiotensin II receptor blocker; ASMD = absolute standardized mean difference; MI = myocardial infarction; PPCI = primary percutaneous coronary intervention; STEMI = ST-elevation myocardial infarction. | | | | | | | |
