# Supplementary material for: All-cause mortality and the risk of stroke with selective aspiration thrombectomy in patients with ST-elevation myocardial infarction undergoing primary percutaneous coronary intervention: A nationwide retrospective cohort study
Source: Medicine (Baltimore). 2020 May 29;99(22):e19590. doi: 10.1097/MD.0000000000019590 (PMC12245257; doi:10.1097/MD.0000000000019590)
Supplement: SUPPLEMENTARY MATERIAL [file medi-99-e19590-s003.docx]

Table S3. Impact of hospital volume of primary PCI on the risks of all-cause mortality and stroke in the patients with STEMI treated with aspiration thrombectomy vs. PCI alone

|  | Volume | Events | (S-)HR (95% CI) | P value |
| --- | --- | --- | --- | --- |
| Hospitalization | Lowest tertile (32.4%) | All-cause mortality | 0.84(0.54-1.29) | 0.417 |
|  |  | Stroke | 1.06(0.21-5.24) | 0.945 |
|  | Middle tertile (34.48%) | All-cause mortality | 0.77(0.46-1.3) | 0.333 |
|  |  | Stroke | 1.12(0.21-5.9) | 0.898 |
|  | Highest tertile (33.12%) | All-cause mortality | 1.03(0.71-1.51) | 0.862 |
|  |  | Stroke | NA | NA |
| Post-discharge | Lowest tertile (31.9%) | All-cause mortality (30 days) | 1.06(0.39-2.88) | 0.908 |
|  |  | Stroke (30 days) | NA | NA |
|  |  | All-cause mortality (1 year) | 0.91(0.51-1.62) | 0.754 |
|  |  | Stroke (1 year) | 0.24(0.03-2.13) | 0.201 |
|  | Middle tertile (35.04%) | All-cause mortality (30 days) | 1.02(0.41-2.58) | 0.959 |
|  |  | Stroke (30 days) | NA | NA |
|  |  | All-cause mortality (1 year) | 0.91(0.5-1.67) | 0.769 |
|  |  | Stroke (1 year) | 0.92(0.24-3.6) | 0.91 |
|  | Highest tertile (33.06%) | All-cause mortality (30 days) | 0.49(0.15-1.68) | 0.258 |
|  |  | Stroke (30 days) | 1.28(0.29-5.61) | 0.744 |
|  |  | All-cause mortality (1 year) | 0.59(0.32-1.1) | 0.095 |
|  |  | Stroke (1 year) | 1.87(0.66-5.35) | 0.241 |
| Overall | Lowest tertile (32.4%) | All-cause mortality (30 days) | 0.86(0.58-1.29) | 0.471 |
|  |  | Stroke (30 days) | 0.73(0.16-3.23) | 0.675 |
|  |  | All-cause mortality (1 year) | 0.94(0.68-1.31) | 0.728 |
|  |  | Stroke (1 year) | 0.6(0.2-1.81) | 0.34 |
|  | Middle tertile (34.48%) | All-cause mortality (30 days) | 1.05(0.65-1.68) | 0.846 |
|  |  | Stroke (30 days) | 0.76(0.17-3.38) | 0.714 |
|  |  | All-cause mortality (1 year) | 0.92(0.62-1.35) | 0.664 |
|  |  | Stroke (1 year) | 1.05(0.36-3.03) | 0.928 |
|  | Highest tertile (33.12%) | All-cause mortality (30 days) | 1(0.69-1.45) | 0.99 |
|  |  | Stroke (30 days) | 0.45(0.11-1.75) | 0.247 |
|  |  | All-cause mortality (1 year) | 0.95(0.69-1.29) | 0.731 |
|  |  | Stroke (1 year) | 1.06(0.43-2.57) | 0.905 |

CI = confidence interval; NA = not applicable because of zero events; PCI = percutaneous coronary intervention; STEMI = ST-elevation myocardial infarction; (S-)HR = (sub-)hazard ratio.
