# Supplementary material for: All-cause mortality and the risk of stroke with selective aspiration thrombectomy in patients with ST-elevation myocardial infarction undergoing primary percutaneous coronary intervention: A nationwide retrospective cohort study
Source: Medicine (Baltimore). 2020 May 29;99(22):e19590. doi: 10.1097/MD.0000000000019590 (PMC12245257; doi:10.1097/MD.0000000000019590)
Supplement: SUPPLEMENTARY MATERIAL [file medi-99-e19590-s004.docx]

Table S4. Impact of physician volume of primary PCI on the risks of all-cause mortality and stroke in the patients with STEMI treated with aspiration thrombectomy vs. PCI alone

|  | Volume | Events | (S-)HR (95% CI) | P value |
| --- | --- | --- | --- | --- |
| Hospitalization | Lowest tertile (31.54%) | All-cause mortality | 0.99(0.68-1.45) | 0.956 |
|  |  | Stroke | 0.44(0.09-2.24) | 0.323 |
|  | Middle tertile (34.99%) | All-cause mortality | 0.91(0.56-1.46) | 0.684 |
|  |  | Stroke | 0.32(0.04-2.98) | 0.32 |
|  | Highest tertile (33.47%) | All-cause mortality | 0.79(0.49-1.27) | 0.329 |
|  |  | Stroke | 0.97(0.2-4.71) | 0.97 |
| Post-discharge | Lowest tertile (29.84%) | All-cause mortality (30 days) | 1.27(0.5-3.18) | 0.616 |
|  |  | Stroke (30 days) | 0.64(0.05-7.82) | 0.725 |
|  |  | All-cause mortality (1 year) | 1.08(0.62-1.91) | 0.78 |
|  |  | Stroke (1 year) | 0.95(0.2-4.62) | 0.953 |
|  | Middle tertile (35.81%) | All-cause mortality (30 days) | 0.59(0.2-1.75) | 0.344 |
|  |  | Stroke (30 days) | NA | NA |
|  |  | All-cause mortality (1 year) | 0.91(0.5-1.64) | 0.752 |
|  |  | Stroke (1 year) | 1.04(0.3-3.64) | 0.95 |
|  | Highest tertile (34.35%) | All-cause mortality (30 days) | 0.76(0.21-2.74) | 0.674 |
|  |  | Stroke (30 days) | 1.52(0.25-9.16) | 0.647 |
|  |  | All-cause mortality (1 year) | 0.47(0.24-0.94) | 0.033 |
|  |  | Stroke (1 year) | 1.11(0.38-3.27) | 0.845 |
| Overall | Lowest tertile (31.54%) | All-cause mortality (30 days) | 1.08(0.75-1.53) | 0.687 |
|  |  | Stroke (30 days) | 0.32(0.07-1.58) | 0.163 |
|  |  | All-cause mortality (1 year) | 1.04(0.77-1.4) | 0.805 |
|  |  | Stroke (1 year) | 0.67(0.24-1.88) | 0.445 |
|  | Middle tertile (34.99%) | All-cause mortality (30 days) | 0.81(0.53-1.26) | 0.35 |
|  |  | Stroke (30 days) | 0.2(0.02-1.67) | 0.136 |
|  |  | All-cause mortality (1 year) | 0.94(0.66-1.34) | 0.72 |
|  |  | Stroke (1 year) | 0.71(0.25-2.02) | 0.519 |
|  | Highest tertile (33.47%) | All-cause mortality (30 days) | 0.92(0.58-1.45) | 0.716 |
|  |  | Stroke (30 days) | 1.39(0.4-4.81) | 0.599 |
|  |  | All-cause mortality (1 year) | 0.82(0.56-1.21) | 0.316 |

CI = confidence interval; NA = not applicable because of zero events; PCI = percutaneous coronary intervention; STEMI = ST-elevation myocardial infarction; (S-)HR = (sub-)hazard ratio.
